# Supplementary material for: Neocortical inhibitory interneuron subtypes are differentially attuned to synchrony- and rate-coded information
Source: Commun Biol. 2021 Aug 5;4:935. doi: 10.1038/s42003-021-02437-y (PMC8342442; doi:10.1038/s42003-021-02437-y)
Supplement: Supplementary file 2 — Supplementary Material [file 42003_2021_2437_MOESM2_ESM.pdf]

SUPPLEMENTARY MATERIAL

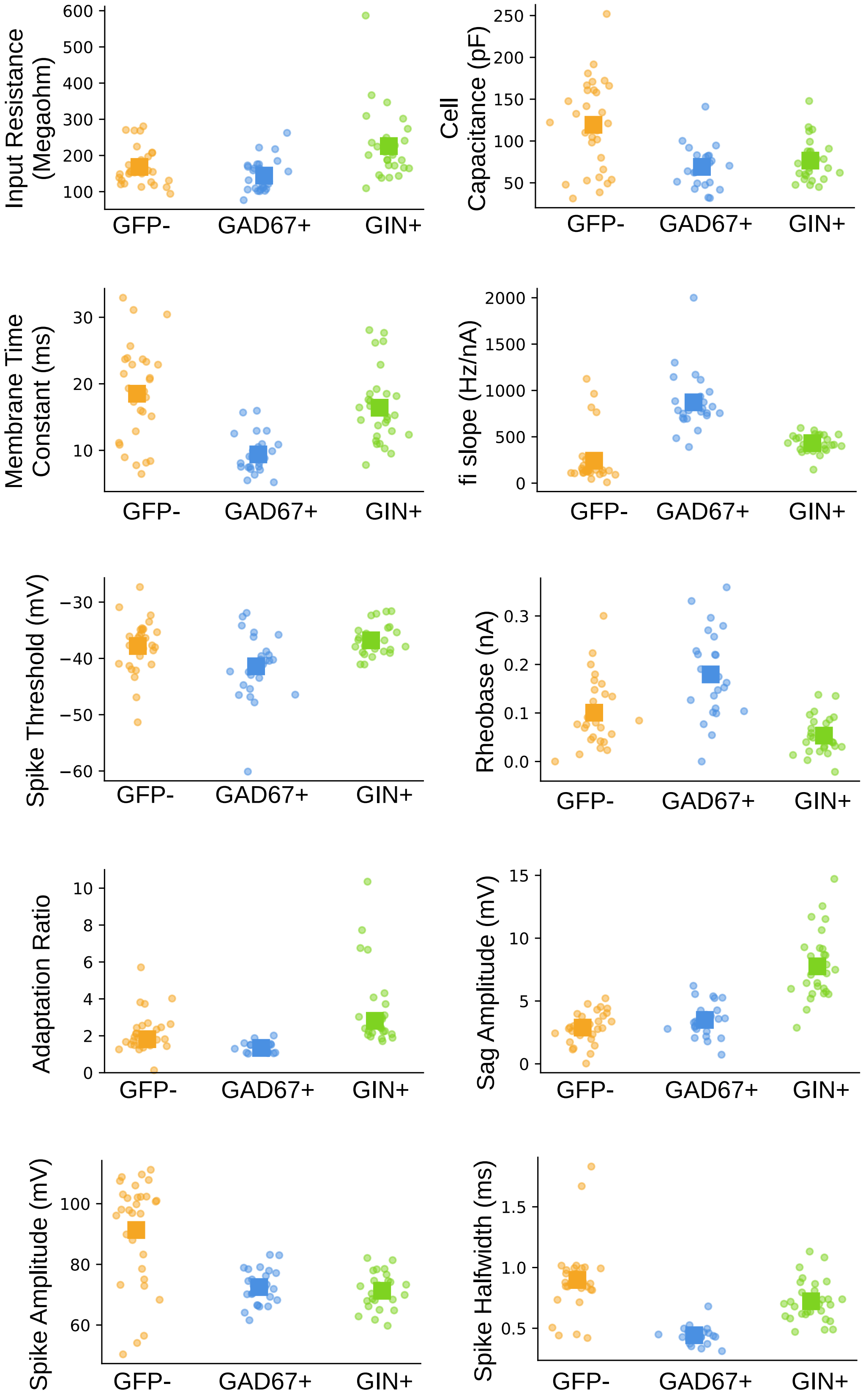
Input Resistance (Megaohm)
600
500
400
300
200
100
GFP-
GAD67+
GIN+
Cell Capacitance (pF)
250
200
150
100
50
GFP-
GAD67+
GIN+
Membrane Time Constant (ms)
30
20
10
GFP-
GAD67+
GIN+
fi slope (Hz/nA)
2000
1500
1000
500
0
GFP-
GAD67+
GIN+
Spike Threshold (mV)
−30
−40
−50
−60
GFP-
GAD67+
GIN+
Rheobase (nA)
0.3
0.2
0.1
0.0
GFP-
GAD67+
GIN+
Adaptation Ratio
10
8
6
4
2
0
GFP-
GAD67+
GIN+
Sag Amplitude (mV)
15
10
5
0
GFP-
GAD67+
GIN+
Spike Amplitude (mV)
100
80
60
GFP-
GAD67+
GIN+
Spike Halfwidth (ms)
1.5
1.0
0.5
GFP-
GAD67+
GIN+

Figure S1: Electrophysiological features estimated from current clamp recordings. Circles indicate estimates from individual cells, squares indicate group means for each cell type. Electrophysiological features can be grouped into four sets 1) Linear filtering (input resistance, cell capacitance, membrane time constant), 2) Activation discontinuity (rheobase, f-I slope), 3) Non-linear filtering (Adaptation ratio), 4) Spike shape (spike threshold, spike amplitude, spike halfwidth). As reported in previous studies, GIN+ cells had higher input resistance, adaptation ratio and sag amplitude than other cell types, in line with their regular spiking phenotype. GAD67+ cells possessed higher rheobase and f-I slope than other cell types, and smaller spike halfwidth and membrane time constant than other cell types, in line with their fast-spiking phenotype. GFP- cells typically possessed higher cell capacitance and spike amplitudes than other cell types, along with lower f-I slopes, traits indicative of L2/3 pyramidal cells.

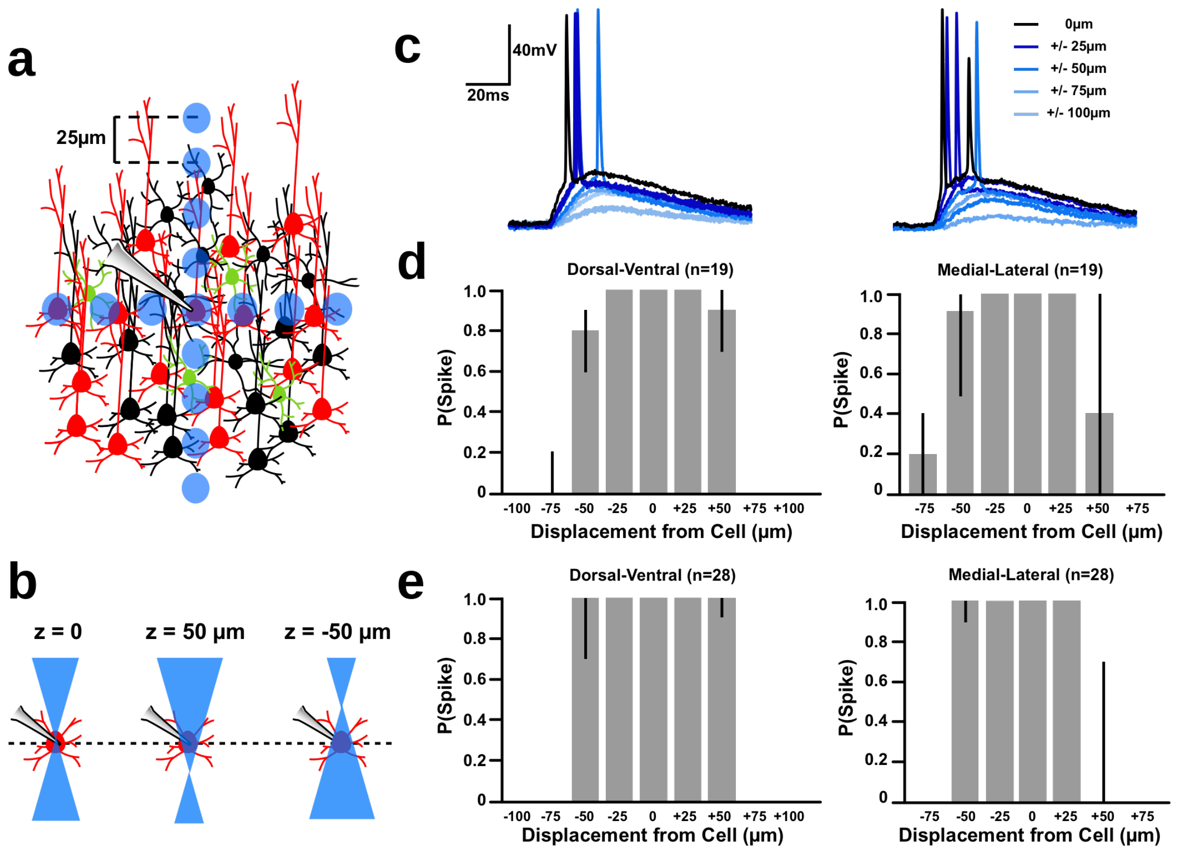

Figure S2: L2/3 Pyramidal Neurons reliably spike when illuminated with 15 µm spots placed ≤ 50 µm away. a) Experimental procedure. b) Given that single-photon illumination produces a cone of illumination above and below cells being stimulated, we conducted the protocol shown in a), while also stimulating the recorded cell when the light was in focus and directly on top of the cell (z = 0), above it (z = 50 µm) or below it (z = -50 µm). c) Sample responses from ChR2+ neuron to spots placed at varying locations when the light was directly focused on the cell (z = 0). d) Median spiking probability of ChR2+ neurons (n= 19) to spots placed at varying locations within the microscope's field of view with spot directly in focus of the cell (i.e. z = 0). e) Same analysis as d) except with cellular responses to spots illuminated when the focal point was above or below the cell of interest (i.e. z = 50 µm or z = -50 µm, n= 28).

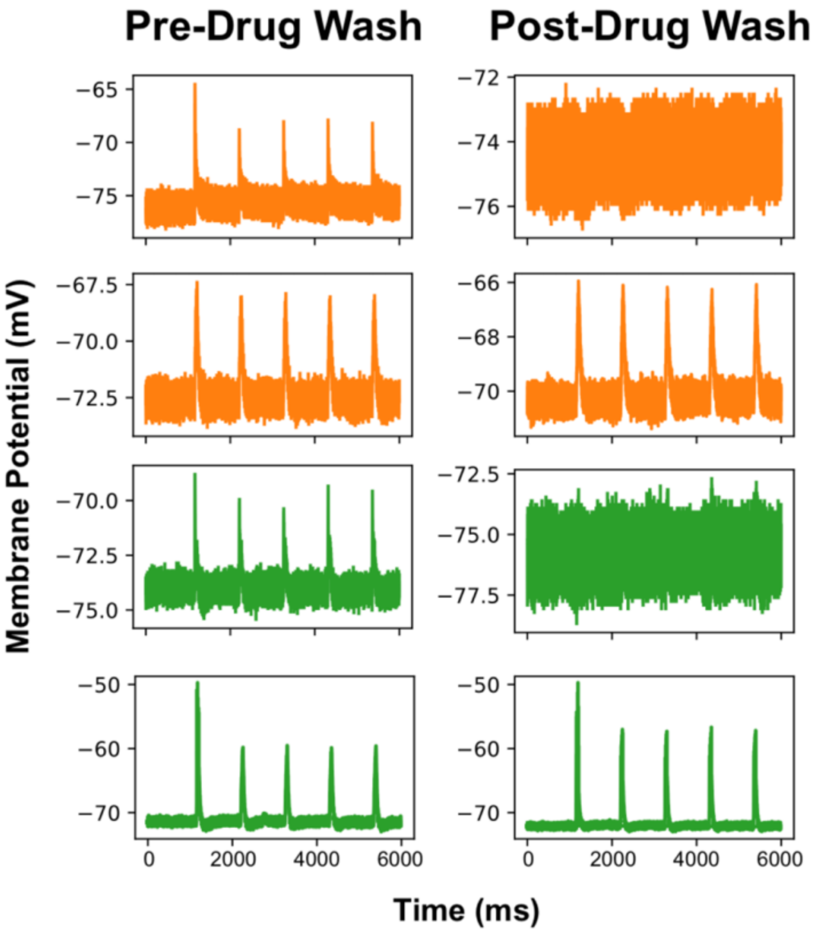

Figure S3: Bath application containing 10 µM of selective NMDA receptor antagonist d-APV and 50 µM of AMPA receptor antagonist NBQX did not reliably block ChR2-mediated responses during 5 bouts of light presentation in mCherry- cells. First and second rows: Recording of an mCherry-GFP- neuron pre and post drug wash. Third and fourth rows: Recording of an mCherry-GFP+ neuron pre and post drug wash. Note that some cells (first and third rows) responded to the drug wash suggesting that they likely did not confer any non-specific ChR2 expression whereas other cells did (second and fourth rows). In these recordings, n = 4 (a trace from each neuron is shown), with mCherry- neurons were selected randomly.

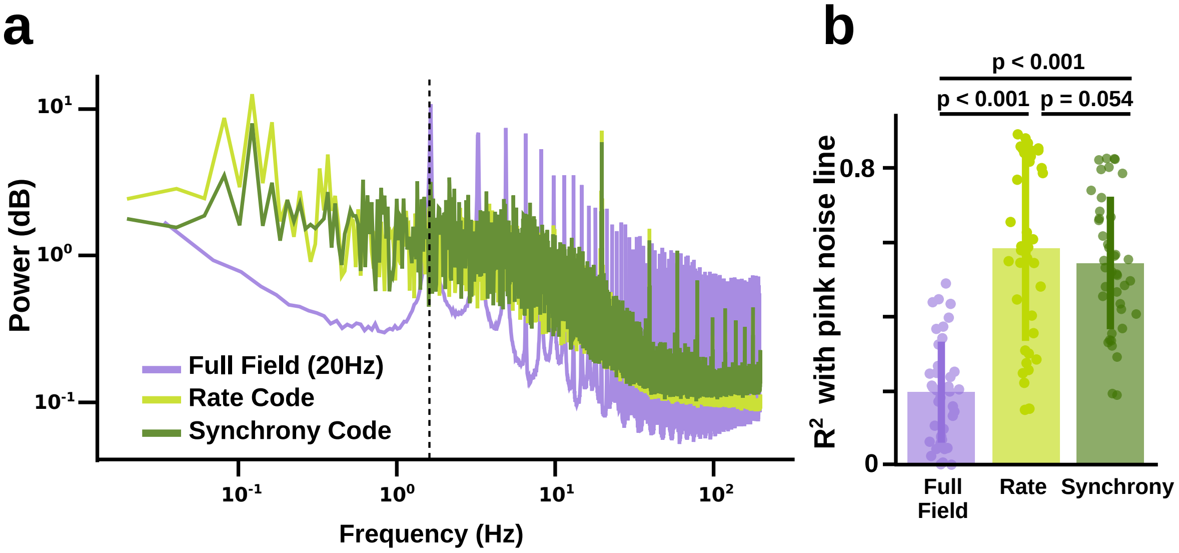

Figure S4: Artificial rate and synchrony of optical inputs to Layer 2/3 pyramidal neurons mimic in vivo-like responses in postsynaptic cells. a) Power spectral density (PSD) of neuronal recordings to either full field (n= 42), rate (n=42) or synchrony (n=42) of optical inputs. Dotted line marks 20Hz frequency. b) Correlations of each spectrum to a pink noise line. (Paired t-tests, Full vs. Rate: $t(82) = -8.65$, $p \leq 0.001$*, Full vs. Synchrony: $t(82) = -9.71$, $p \leq 0.001$*, Rate vs. Synchrony: $t(82) = 2.067$, $p = 0.054$, * = tests significant at $p \leq 0.017$ with Bonferroni correction).

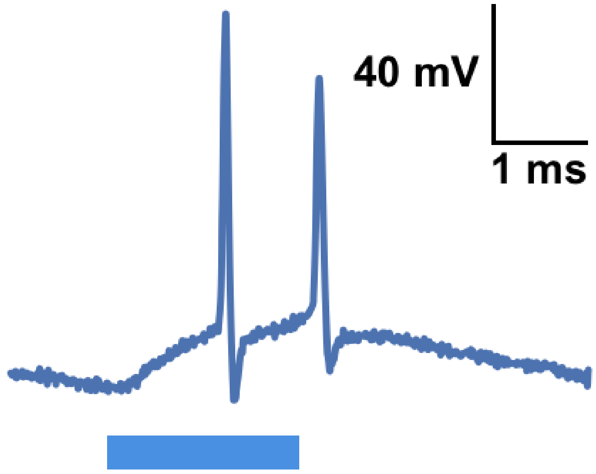

Figure S5: Prolonged polysynaptic activity was produced upon activation of our ROIs.

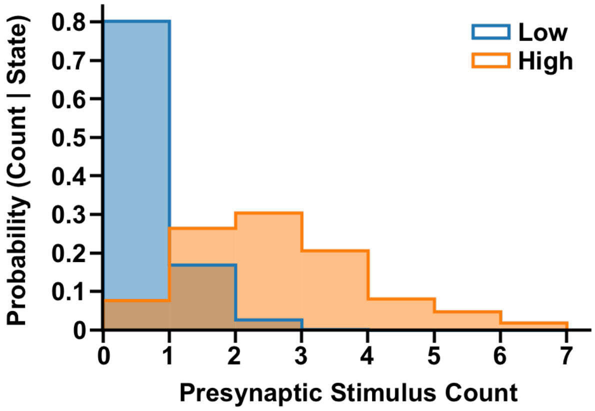

Figure S6: Presynaptic spike count increased when higher rates of optical input were used.

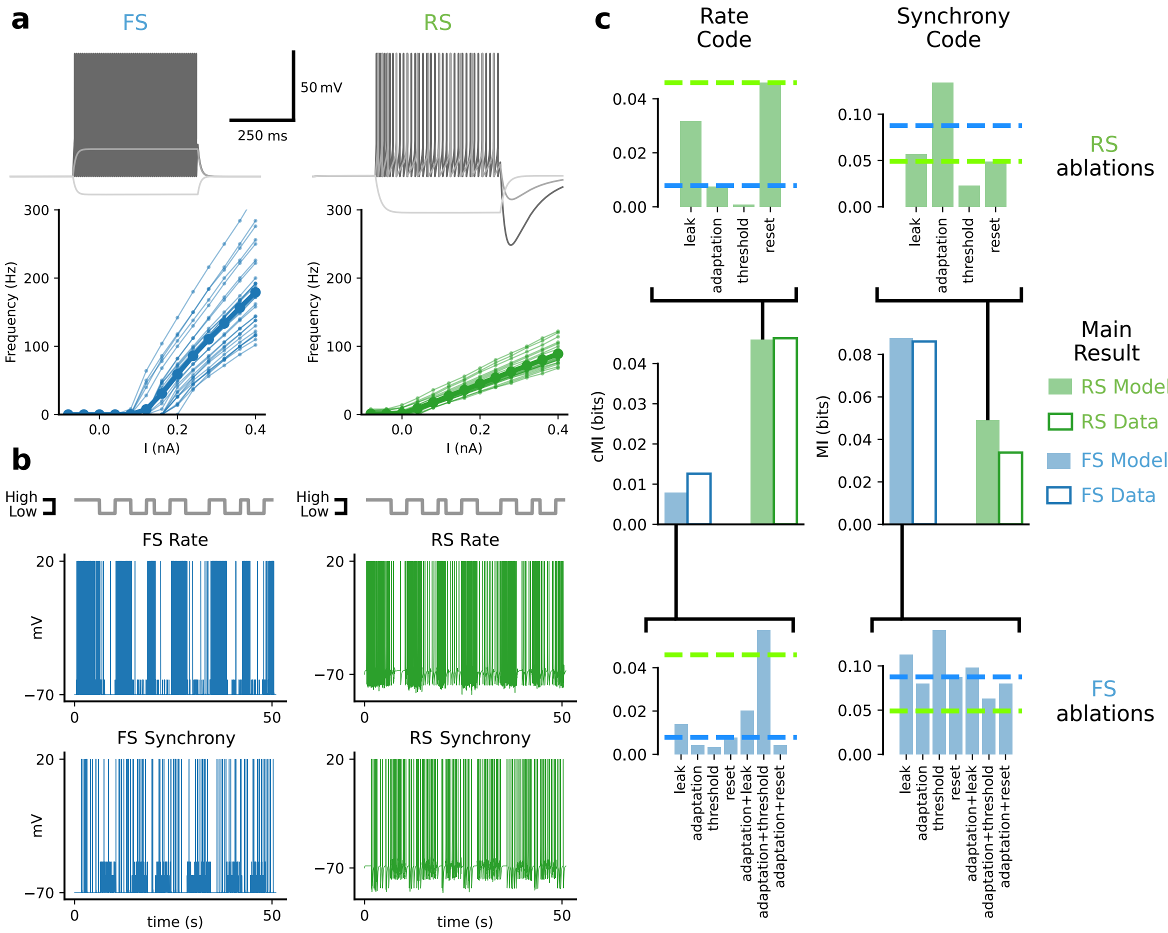

Figure S7: Reproduction of experimental findings with AdExp neurons. a) Current injection simulations for FS (left) and RS (right) interneurons. Above: sample traces at -0.08 pA (light grey), 0.12 pA (medium grey), 0.32 pA (dark grey). Below: individual (light lines) and mean (heavy lines) f-I curves. b) Example membrane potential traces in response to rate (top) and temporally (bottom) coded stimuli. Coded 1-bit signal shown in grey (above). c) Middle: Mutual information between post-synaptic spike counts and 1-bit signal for rate code (left; conditioned on pre-synaptic spike count) and temporal code (right). Filled= simulation, Unfilled= experiment. Insets show parameter ablations for RS (above) and FS (below) interneurons. Dashed blue lines correspond to unablated MI for FS, dashed green lines correspond to unablated MI for RS.

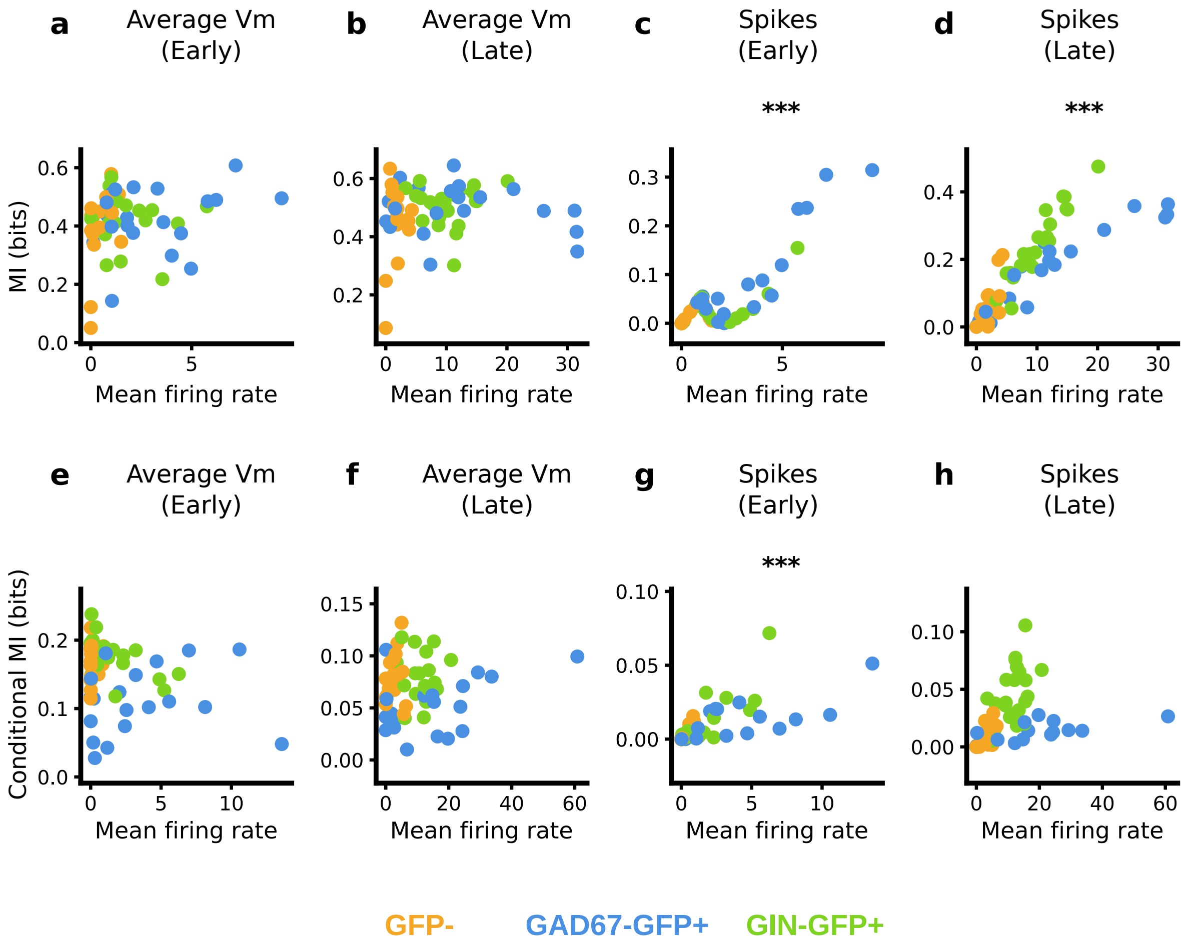

Figure S8: Relationship between mutual information and average firing rate. a) Correlation between mutual information between the average membrane potential and the synchrony code in the early window and mean firing rate of the post-synaptic neuron during the synchrony code protocol. b) Same as A) but in the late window. c) Same as a) but for MI between post-synaptic spike counts in the early window and synchrony coded signal. d) Same as c) but in the late window. e-h) Same as a-d) but for conditional mutual information with the rate coded signal. Stars indicate Bonferroni corrected significance at p<0.0001

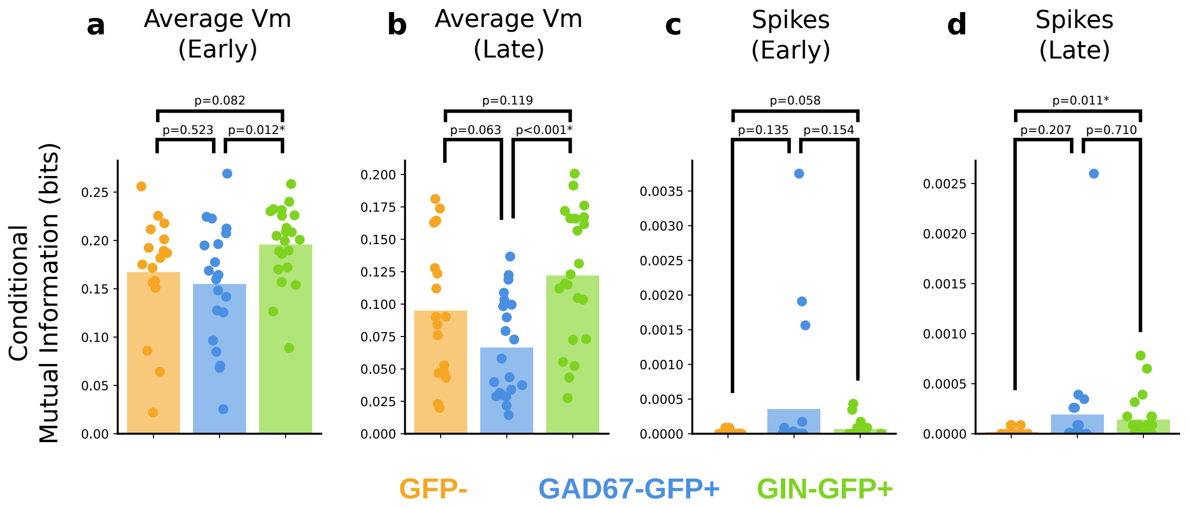

Figure S9: Conditional mutual information analysis of the average membrane potential in the early (a) and late windows (b) to the synchrony coded response. c-d): Same as a-b), but for spike counts. Comparisons indicate post-hoc t-tests, with significance at $p < 0.017$.

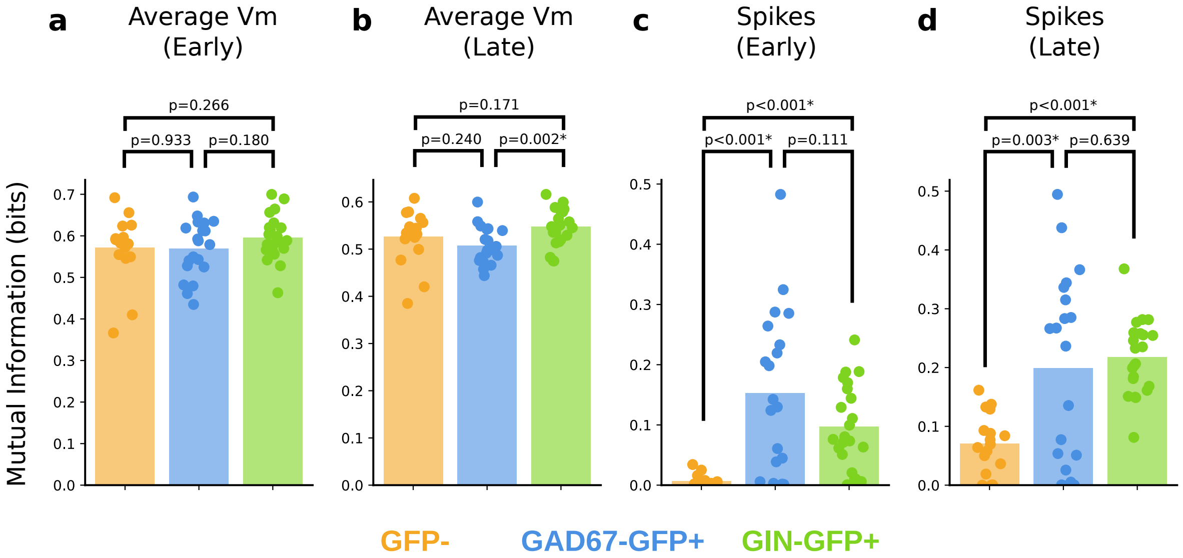

Figure S10: Mutual information analysis of the average membrane potential in the early (a) and late windows (b) to the rate coded response. c-d): Same as a-b), but for postsynaptic spike counts. Comparisons indicate post-hoc t-tests, with significance at $p < 0.017$.
